# Tracing the indirect societal impacts of biomedical research: development and piloting of a technique based on citations

**DOI:** 10.1007/s11192-016-1895-4

**Published:** 2016-03-08

**Authors:** Teresa H. Jones, Steve Hanney

**Affiliations:** Health Economics Research Group, Brunel University London, Uxbridge, Middlesex UB8 3PH UK

**Keywords:** Citation categorisation, Societal impacts of research, Qualitative analysis, Citation generations, Research assessment

## Abstract

There is growing interest in assessing the societal impacts of research such as informing health policies and clinical practice, and contributing to improved health. Bibliometric approaches have long been used to assess knowledge outputs, but can they also help evaluate societal impacts? We aimed to see how far the societal impacts could be traced by identifying key research articles in the psychiatry/neuroscience area and exploring their societal impact through analysing several generations of citing papers. Informed by a literature review of citation categorisation, we developed a prototype template to qualitatively assess a reference’s importance to the citing paper and tested it on 96 papers. We refined the template for a pilot study to assess the importance of citations, including self-cites, to four key research articles. We then similarly assessed citations to those citing papers for which the key article was Central i.e. it was very important to the message of the citing article. We applied a filter of three or more citation occasions in order to focus on the citing articles where the reference was most likely to be Central. We found the reference was Central for 4.4 % of citing research articles overall and ten times more frequently if the article contained three or more citation occasions. We created a citation stream of influence for each key paper across up to five generations of citations. We searched the Web of Science for citations to all Central papers and identified societal impacts, including international clinical guidelines citing papers across the generations.

**Electronic supplementary material:**

The online version of this article (doi:10.1007/s11192-016-1895-4) contains supplementary material, which is available to authorized users.

## Introduction

The importance of assessing the impact and translation of biomedical research is increasingly being recognised by research funding bodies, partly as a way of demonstrating accountability for their spending to taxpayers and charitable donors. Recent reviews in the health field have identified the growing interest in expanding the scope of research evaluation so that in addition to assessing knowledge production it also covers economic/societal (or wider, or non-academic) impact of research in terms of informing health policies and clinical practice, and generating health and economic gains (Banzi et al. 2011; Bornmann 2013; Milat et al. 2015). Such reviews often also found the Payback Framework (Buxton and Hanney 1996; Donovan and Hanney 2011) to be the most frequently used approach to assess health research impact. One of the prominent features of the Payback Framework is its multi-dimensional categorisation of benefits. This includes the full range of impacts starting with knowledge production which can be assessed by traditional bibliometrics and, for example, was done so extensively in a study of the impacts of the National Breast Cancer Foundation (NBCF) in Australia (Donovan et al. 2014).

There are many well-established ways of conducting bibliometric analysis, and new approaches are frequently proposed (Wu 2015), but it is widely recognised that it is more difficult to assess outcomes, such as impacts on health, than outputs such as publications (Weiss 2007). Methods to assess outcomes are much less well developed. Nevertheless, there has been considerable recent progress reported in applications of the Payback Framework, and other studies reported in the reviews described above (Banzi et al. 2011; Milat et al. 2015). Furthermore, in the UK the Research Excellence Framework (REF) 2014 was successfully applied to assess the quality and impact of research from all UK Higher Education Institutions (Higher Education Funding Council 2015).

In this article we shall focus specifically on societal impacts, which the UK Medical Research Council (MRC) define as: ‘Increasing the effectiveness of public services and policy. Enhancing quality of life, health and creative output.’ (MRC 2015). There has been some interest in exploring how far approaches such as citation analysis that are used for assessing outputs can be extended and also be used to assess societal impacts. Some early studies started by looking at the role of citations in clinical guidelines (Grant et al. 2000) or in items that could be part of the societal impact achieved by health research such as other policy documents or text books (Lewison 2004). These early explorations are now being supplemented by further attempts to establish the scope of such approaches and consider how far they can demonstrate the societal impact from the research of particular funders or research centres (Kryl et al. 2012; Sullivan et al. 2011). The grey literature is also an increasingly important source for searches of citations on health policy documents (Sibbald et al. 2015). Furthermore, in some payback studies attempts have been made not just to identify the clinical guidelines on which research papers from a particular funder might be cited, but also to go further in detailed case studies and consider the importance of papers from a funder, such as Asthma UK, in terms of the specific contribution the paper might make to a particular guideline. This can be undertaken by considering factors such as: the importance of the point being made in the guideline that the citation is being used to support; how far that reference is the major source of evidence supporting the point in the guideline; and whether or not a paper is cited more than once on a particular guideline (Hanney et al. 2013).

So, new forms of citation analysis definitely have a role to play in assessing the societal impacts from research. There are, however, important limitations, including that such analysis of citations usually only focuses on the direct influence from the paper cited, and it is argued that impacts usually arise from one or more streams of research or from a variety of papers.

Some researchers have already advocated that standard bibliometric analysis should consider more than one generation of citations by examining the inclusion of indirect as well as direct citations (Dervos and Kalkanis 2005; Fragkiadaki and Evangelidis 2014; Hu et al. 2011; Rousseau 1987). In 1987 Rousseau proposed the Gozinto theorem to determine the cited references that had the greatest influence on the paper under scrutiny. As part of his mathematical calculation of the total influence of the referenced paper, Rousseau added weights to the direct and the indirect references. He considered his method could equally be used looking forward to study the chosen paper’s direct and indirect citations or backward to study direct and indirect references. Hu et al. (2011) similarly considered that direct citations to a publication only told part of the story of the contribution made by a paper to the evolution of its field of research and to science in general. They concluded that by taking more than one generation of citations into account the structure of the network underlying the progress in science could better be revealed. Dervos and Kalkanis (2005) included more than one citing generation in their framework for calculating the bibliometric impact of a research paper as they considered it provided a fairer quantitative method of assessment because it also took into consideration the research activity that had been triggered by the original publication. Fragkiadaki and Evangelidis (2014) reviewed citations across more than one generation (direct and indirect citations) and provided an overview of the concepts of multiple generations of citations and the indirect impact.

Kostoff (1998) commented that ‘one largely unutilised role of citations is to serve as a “radioactive tracer” of research impacts…this is a very fruitful area for future citation research and analysis’. So, would it be feasible to indeed use citation analysis through a series of generations of papers in order to contribute to an analysis of the societal impacts from health research by examining whether later generations of citing papers were cited on documents such as clinical guidelines? In the account described here we adopt the term ‘generations of citing papers’ to mean moving from a source paper to the citing papers, and then to the papers that cite the citing papers and so on.

To be able to conduct such analysis we believe we would have to address a range of overlapping issues each of which have sometimes been explored, but which we do not believe have previously been brought together to address the concept of using citation analysis to trace the indirect societal impact of health research over a longer perspective. There are questions of the legitimacy and practicality of organising citation analysis through several generations, and taking account of not just direct citations but also of what some authors have described as ‘indirect citations’. As Rousseau (1987) noted, the numbers of papers involved could rapidly become very large. But many citations are known just to be perfunctory (Kacmar and Whitfield 2000; Prabha 1983; Safer and Tang 2009), therefore any such exercise would soon risk considering large numbers of perfunctory citations of papers that themselves have perfunctorily cited the source paper, and so on. There could be large numbers of papers with a minimal connection to the original source paper. So, the questions of legitimacy and practicality overlap. Almost 30 years ago, Rousseau (1987) developed a formula that would take into account the fact that not all citations are of equal importance. But given the scale of the analysis envisaged it was suggested that this should be addressed in a formulaic way by, for example, giving citations different weights according to their location in the citing paper. This approach may be appropriate at the level of broad generalisations, but when the question being addressed is whether it is possible to identify the main references in order to selectively follow through from generation to generation then the ability explicitly to identify the most influential individual references becomes very important.

In 2000, Kacmar and Whitfield (2000) assessed the influence of a body of papers from two journals (*AcademyofManagementReview* and *AcademyofManagementJournal)* on the papers citing them. They studied each citing paper to determine whether the reference was a major basis for the paper. They found that the papers from the *AcademyofManagementReview* were important for only 9 % of citing papers and papers from the *AcademyofManagementJournal* were important for only 6 % of citing papers. In 2005, Hanney et al. developed a method to identify a wide range of impacts from a body of research by using a variety of both qualitative and quantitative techniques. One such technique involved categorising citations received by a paper to identify those where the reference was considered important (Hanney et al. 2005). The analysis looked at a second generation consisting of all the papers citing 29 first generation papers. They concluded that the number of citing papers for which the reference was at least of considerable importance was only 9 % of the total number of citations, with just 1 % being essential (Hanney et al. 2005). This means that in any major analysis through generations of citing papers the identification of the citing papers for which the reference was highly important is a crucial practical step and, further, that this could require a multi-step assessment process.

We aimed to build on previous work on qualitative citation analysis in order to explore the development of biomedical research over numerous generations of citations, and eventually to identify indirect societal impacts. We had previously examined the literature in order to help us develop a robust citation categorisation method for application to biomedical research in order to help explore the societal impacts from that research (Jones et al. 2012). We had searched the literature extensively including by: automated searches of citation databases and subject specific databases; manual searches of four prominent journals: and by using ‘snowballing’ techniques on eight key papers. We identified and systematically reviewed 9050 potentially relevant papers and examined 285 in detail including many examples of studies discussing the meaning or use of citations and also papers discussing the importance within a paper of different citations. In the literature survey we identified both objective and subjective elements (i.e. independent and dependent on the assessors’ judgement) that could potentially help us to identify the citations for which the reference was very important.

In our continued monitoring of the literature we have identified further developments since our review published in 2012. In a recent review, Ding et al. (2014) gave an overview of content-based citation analysis which they considered to be the next generation of citation analysis, concluding that the development of a way to give weight to important citations within a citing paper was one of the tasks still unanswered (Ding et al. 2014). Zhu et al. (2015) built on previous work and claimed that the number of times a reference is mentioned in the body of a citing paper was one of the best ways of measuring academic influence.

In this article we describe the development and refinement of a qualitative citation assessment procedure that incorporated both objective and subjective elements identified in our literature survey (Jones et al. 2012). We developed a prototype template for the categorisation of citations, and tested it on a small number of papers. We then reflected on the results, revised the template and piloted it. We also applied an initial filter that reduced the number of papers we had to consider. In this pilot we aimed to trace the citation streams of some chosen health research articles across up to six generations of citations in order to understand more fully the progress and development of the research, and to explore the potential for using the method as a contribution to the identification of the societal impacts of the original health research. Considerable progress was made in the pilot and in the “Discussion” section we consider how far this new approach could contribute to the increasing focus on assessing the societal impacts of research.

## Method

We developed a prototype template, tested it on a group of 96 papers and modified the template based on our findings for use in the pilot. The modified template was applied to the papers that cited a small group of chosen key research articles. We used the papers for which the key research article was important and examined the citations to those papers through several generations to identify the indirect societal impacts of the research. Below we set out the full sequence of activities we undertook first in the testing of the prototype and then in the pilot and the search for societal impacts.

### Description and testing of the prototype template developed as a result of the literature search

Following our literature search on qualitative citation analysis (Jones et al. 2012) we used the gathered evidence to inform the development of an assessment procedure for the qualitative citation analysis of biomedical research papers. We constructed a prototype assessment template that consisted of three sections: the first section collected some preparatory details; the second section related to characteristics of citations within a paper for each and every occasion where the reference was cited; and the third section considered the relationship between the reference and the citing paper as a whole including the way in which the reference was used within the citing paper and its importance to the citing paper. A space for comments and an instruction sheet for further guidance were also provided (Online resource 1).

To test the prototype template we applied it to a body of mental health research covering basic neuroscience as well as community psychiatry and clinical psychology as two ends of a spectrum of neurological/mental disorders. The mental health field was chosen for this study as interesting comparisons could potentially be made with findings from other studies on the impact of such research especially the Mental Health Retrosight project (Wooding et al. 2014a, b).

We identified research articles authored by two prominent researchers, Professor Tim Bliss researching an area of neuroscience and Professor Paul Salkovskis researching an area of clinical psychology. A selection of 96 research articles and reviews referencing articles authored by either Bliss or Salkovskis were chosen for study. Previous research had found that references cited on more than one occasion within a paper indicated greater influence of the reference to the citing paper (McCain and Turner 1989; Peritz 1983; Safer and Tang 2009; Sombatsompop et al. 2006; Tang and Safer 2008), therefore we preferentially selected papers with more than one citation occasion to increase the chances of including more papers where the reference was of high importance. Our selection of papers included 19 % of citing research articles with three or more citation occasions and 52 % of citing reviews with two or more citation occasions. Otherwise the selection was random from those papers identified on Web of Science (WOS) and listed in date order and available in full via Brunel University library.

A number of experts (Online resource 2) were invited to participate in the research project to provide guidance throughout. This group consisted of experts in social psychiatry, community psychiatry, neuropsychiatry, health and research policy and research impact.

We advertised for post-graduate students to carry out the assessment of the citations specifying our requirements for: a high level of ability in written and spoken English; a good understanding and preferably a working knowledge of the structure of scientific published papers; at least a graduate level of education; and availability for the whole time-frame of the assessment process. Previous knowledge of the area of research being studied in the project was not looked for in the selection of reviewers. Introductory sessions were held for the successful applicants together with group training sessions and practice papers. Group discussion sessions were also held to deal with differences in understanding of the evaluation procedure that was to be used and additional contact was provided when it was considered beneficial to the reviewers’ understanding of the evaluation process and to consistency in its application.

In preparation for the assessment process, the full papers (preferably in pdf form) were obtained either electronically or as paper copies from Brunel University library. Adobe Acrobat 9.0 was used to find and highlight the reference in the bibliographies of the pdf copies as well as the locations of the citation occasions to that reference. Bibliographic details of the cited and citing papers as well as details for each citation occasion were pre-entered on the assessment template before its distribution together with a highlighted copy of the paper for assessment. In total there were 15 assessors (4 subject experts and 11 reviewers including 8 post graduates, 2 researchers i.e. TJ and SH, and a bibliometrics expert who became a research team member i.e. CD). They each assessed all papers. Completed assessment sheets were returned to the researchers for analysis using Microsoft Excel.

The discussion in the literature (Jones et al. 2012) about the pros and cons of inclusion of self-citations in citation analysis was inconclusive for our purposes. Therefore, we took the decision to include self-citations and collect data on them in the same way as for other citations. We would then be in a more informed position to examine the findings and make comparisons.

The nature of citations in reviews was also considered potentially to be different to citations in research articles and therefore a slightly different template was created for application to reviews.

### Refinement of the prototype template for application in the pilot

The data collected in the testing of the prototype template (Online resource 2) were presented and discussed at a meeting with the panel of experts. We found that the opinions of the assessors on the use of the references by the citing authors were too varied for use as a categorisation procedure that might inform tracing the influence of a research article across several citation generations. Following much discussion, the meeting concluded that what was ultimately required from this assessment procedure was identification of the citing papers where the reference played a very important role rather than the identification of the type of role played. Therefore the most helpful part of the prototype template was a single question regarding the importance of the cited article to the citing article. For our purposes this was considered to be the principal question.

The meeting considered further that our principal question and accompanying guidance notes required re-wording to increase clarity on the decision that was to be made. We restructured the principal question and guidance notes with input from all experts and researchers and agreed upon the question:

“Is the cited article CENTRAL to the message of this paper?” This had the following supporting guidance: Tick yes if the KEY CONCLUSIONS of this paper as a whole could not have been reached without one or more of the following:

- By applying a novel theory, method, scale or technology, etc. set out in the cited article.
- By supporting or developing, either by modification or different application, a concept or method set out in the cited article.
- By refuting a concept or method from the cited article.

The assessors were asked to adopt a default position of ‘NO’ when answering the question. [We also concluded that establishing a middle category was desirable. If the answer to the first question was NO then the assessors were asked to also consider a second question that would provide a middle category (Online resource 1)].

In our literature review (Jones et al. 2012) we had found that the citing papers where the reference was very important was likely to be a small percentage of all citing papers and so it would be beneficial to include an instrument in the assessment procedure that would help to focus the assessment on those papers where it was more likely that the reference would be viewed as Central, if such an instrument was available. The majority of the objective data that we collected in the prototype phase were insufficiently definitive for this purpose but we had found that within all research articles where the reference was considered to be important by at least one expert, the reference had been cited on three or more occasions. We therefore decided to introduce an initial filter of at least three citation occasions within a citing research article into the pilot, and, as a check on this filter, we decided to also assess 20 % of the excluded papers.

We also concluded that the role of references in reviews remained uncertain in the context of this assessment process and different from that of research articles, and that this role was important for the transfer of knowledge forwards towards clinical practice. Therefore the assessment procedure for reviews should be considered separately, and in light of the very limited results found in the testing of the prototype template should perhaps be more inclusive and with a different procedure employed. Therefore, due to this uncertainty, and as there were likely to be fewer reviews for assessment, the meeting decided to include all reviews with two or more citation occasions. This initial filter would be followed by just one question to determine a level of importance that required inclusion in the next round of citation analysis. The question was:

“Is the cited article IMPORTANT to a key message from this review/discussion paper?” with a default position of NO. We again provided additional guidance: “Tick yes if the cited article is used to help reach or sustain a KEY TAKE-HOME MESSAGE or CONCLUSION of this review/discussion paper i.e. there is one or more citation occasion which describes the cited paper in some detail (likely to be at least one full sentence) *AND* that or another citation occasion occurs at a point in the text where a key conclusion or take-home message from the review is being developed or discussed)”.

As a check, again we decided to assess 20 % of excluded reviews. The final templates used in the pilot, named the HERG Assessment of Citations Template (HACT) can be found in Online resource 1.

A further point that needed consideration was the role of self-citations in comparison to non-self-citations when the assessment was qualitative. Relevant papers had been identified in our literature search (Jones et al. 2012) and we found that the inclusion of self-citations in assessments has been a much discussed issue by researchers (Hartley 2012; Harzing 2010; Kacmar and Whitfield 2000; Prabha 1983; Safer and Tang 2009; Snyder and Bonzi 1989; Tang and Safer 2008). As we had insufficient evidence to exclude self-citations we took the opportunity to examine this issue in the context of our evaluation procedure. There are many definitions of ‘self-citation’ and as WoS was being used to detect citing papers then we used the definition provided by and used by WoS. (“Self-citations refer to cited references that contain an author name that matches the name of the author of a citing article i.e. an author cites an earlier published paper that he or she authored”. http://images.webofknowledge.com/WOK45/help/WOS/h_citationrpt.html).

### Selection of the bodies of mental health research to be studied

We were looking for work published about 10–15 years previously (approximately 1995–2000) as a result of research funded as part of a specific funding initiative or programme in the area of Mental Health. A short-list of five bodies of work was put to the meeting and prioritised. We chose five articles from these areas, selecting by using methods such as citation counts and publication in journals that were highly respected by clinicians (Jones et al. 2004) in order to maximise our chances of studying work that had led to societal impacts. Inclusion of our initial filters that focused assessment on selected citations meant that the numbers of key articles that we could process, while still uncertain at the start of the pilot, was more than it would have been. So, we prioritised five such articles rather than just considering two articles as had been our original intention.

The chosen bodies of research, in priority order were:

1. NHS R&D Programme: Mental Health and Learning Disability
  - Kuipers, E., Garety, P., Fowler, D., Dunn, G., Bebbington, P., Freeman, D., et al. (1997). London-East Anglia randomised controlled trial of cognitive-behavioural therapy for psychosis. I: effects of the treatment phase. *TheBritishJournalofPsychiatry*, *171*(4), 319–327.
  - Burns, T., Creed, F., Fahy, T., Thompson, S., Tyrer, P., & White, I. (1999). Intensive versus standard case management for severe psychotic illness: a randomised trial. *TheLancet*, *353*(9171), 2185–2189.
2. Vesa, J., Hellsten, E., Verkruyse, L., Camp, L., Rapola, J., Santavuori, P., et al. (1995). Mutations in the palmitoyl protein thioesterase gene causing infantile neuronal ceroid lipofuscinosis. *Nature*, *376*(6541), 584.
3. Clark, D. M., Salkovskis, P. M., Hackmann, A., Middleton, H., Anastasiades, P., & Gelder, M. (1994). A comparison of cognitive therapy, applied relaxation and imipramine in the treatment of panic disorder. *TheBritishJournalofPsychiatry*, *164*(6), 759–769.
4. Richardson, W. D., Pringle, N., Mosley, M. J., Westermark, B., & Dubois-Dalcg, M. (1988). A role for platelet-derived growth factor in normal gliogenesis in the central nervous system. *Cell*, *53*(2), 309–319.

### Preparation of the citing papers

The preparation of the citing papers before assessment was carried out by TJ and EN as had been previously carried out in the testing of the prototype template. Reviews were automatically identified and prepared separately from research articles. For research articles, if three or more citation occasions were identified then processing of the paper was continued. For reviews, processing was continued when two or more citation occasions were identified. In addition, for both research articles and reviews, every fifth and nearest to fifth available paper in time order, from those remaining, was processed. This provided the 20 % sample of those excluded by the filter. Where there were fewer than ten remaining citing papers then two such citing papers were processed. These measures of inclusion resulted in a larger than 20 % sample of articles with fewer than three citation occasions and reviews with fewer that two citation occasions being analysed.

The processed documents were carefully labelled to ensure identification with the correct citing generation as well as with the correct reference.

### The assessment procedure

A secure online application was constructed specifically for this assessment process. This allowed: the central up-loading of previously prepared batches of papers; secure access to the papers and assessment template by the assessors for assessment; electronic recording of the assessment results by the assessors; and batch collection of results to be carried out centrally. New batches of processed papers were uploaded regularly and the assessors were asked to complete their assessment within a fixed time scale. Once the assessment of a paper had been completed and submitted by an assessor then that assessment form was no longer available to that assessor.

Eight assessors who had joined us for the testing of the prototype template were introduced to the refinements that had been made and given some practice papers to help them prepare for the pilot. A discussion session was held to help address any uncertainties.

Part of the way through the assessment process, two of the assessors were unable to continue and had to leave the project thus reducing our numbers to six assessors. We adjusted our assessment procedure to accommodate this change by asking one of the researchers (TJ) to carry out the extra assessments required. This allowed us to maintain the same number of assessments per paper.

The assessment of the citing paper either as Central for research articles or Important for reviews proceeded as follows:

1. Where three or four assessors out of four considered the reference to be Central/Important then the citing paper was taken through to the next citation generation.
2. Where two assessors out of four considered the reference to be Central/Important then the citing paper was additionally assessed by two researchers and if either considered it Central/Important then it continued through to the next citation generation.
3. Where just one or no assessors considered it to be Central/Important then the paper was not studied further.

### Analysis of the results and applying the template to further generations of papers

The centrally collected results were regularly downloaded to an Excel spreadsheet for analysis and to inform the preparation of the next generation of citing papers. We called the key articles generation 1, generation 2 consisted of all the papers citing the four key articles. All of these citing articles where the key article was assessed as Central, and reviews where the key article was assessed as Important, we called generation 2 (Central). Generation 3 consisted of all citing papers to generation 2 (Central). We prepared and assessed them in the same way as described above to create generation 3 (Central). Therefore, generation 3 (Central) consisted of all of the citing articles where a generation 2 (Central) paper was assessed as Central and all reviews where a generation 2 (Central) paper was assessed as Important. We continued to study additional generations of citing papers in the same way to create generation 4 (Central), generation 5 (Central) etc. until either there were no more citing papers to be considered, we reached generation 6 (Central) for that key article or we ran out of time.

In order to manage the uncertain numbers of papers that would require assessment, and expecting the numbers of citing papers to increase as we worked through at least the first two or three generations, we started with our first key article (Kuipers et al. 1997) and worked through at least two generations of citing papers to Kuipers et al. (1997) before starting our study of the next key article (Burns et al. 1999) and so on. The result of this process was likely to be that we would not start or make as much progress with our fourth and fifth key articles (Clark et al. 1994; Richardson et al. 1988) as we did with the other three key articles. Final analysis was carried out in Microsoft Excel.

### Experts’ views on a sample of assessed papers

As a form of quality control, a small number of citing papers from across the citing generations to all key articles were selected and distributed to the experts for their assessment. The papers selected included samples of: Central articles with less than three citation occasions; Central articles with three or more citation occasions; non-Central papers; papers that reviewers’ had had particular difficulty assessing; and where there was an issue that was considered important for discussion for example citations included in letters and book reviews. Findings from these assessments were discussed at a meeting of all experts and researchers.

### Identification of societal impacts

We considered the societal impacts of the key research articles that could be explored using citations, and that therefore we might identify using this method, to be any evidence of their use in clinical guidelines or case reports. Case reports are sections in some medical journals where clinicians describe the treatments given in particular, anonymous, cases and where references might be given to show the evidence supporting the particular treatment. We explored the societal impacts of the original research by examining all citing papers to the key research articles and all citing papers to those articles/reviews where the reference was assessed as Central/Important across all citation generations studied. The direct and indirect societal impacts were measured as follows:

- We went back to the WoS but used a different process to that used for preparing the citing papers. Using all key research articles, and the Central/Important papers in all generations of citations that we had examined in our assessment process to each key article, we identified all citing papers from the Web of Science. We collected just the abstracts and transferred them to EndNote. These abstracts were then automatically searched for the terms ‘guideline’ or ‘case’ located anywhere using EndNote’s search facility. The abstracts of the identified papers were studied and when necessary whole papers obtained so that any evidence of use in clinical guidelines and case reports could be found.
- A web-search using Google was carried out on the key articles in order to identify any guidelines or case reports that had directly cited these articles, but resources restricted the extent to which this could be completed.

### Comparison of the finding

We used other methods to compare with our findings. These included requesting authors’ views on the societal impacts of the research described in the key article via a structured questionnaire and the views of experts in the field via a similar structured questionnaire. Details of the findings from these will be reported more fully elsewhere.

## Results

The results of our pilot including the application of the HACT template are included here for four of the key articles (Burns et al. 1999; Clark et al. 1994; Kuipers et al. 1997; Vesa et al. 1995) considered as one body. Time did not allow us to complete the assessment of all papers citing the fifth key article (Richardson et al. 1988) and therefore they have been omitted. The results for the identification of the societal impacts are also discussed.

### Number of assessments

Assessments took place over a 9 month period ending in September 2011. A small number of papers were not available to us over this period for various reasons. However we had included steps in the method to ensure the correct proportions of papers from each category were included. The numbers included and assessed can be found in Table 1.

**Table 1:** Numbers of papers (research articles and reviews) included in the citing streams of all four key articles (generation 1): the numbers assessed and the numbers of those assessed that were considered Central/Important

Number of papers included in study	Total citing	Number assessed	Number Central/Important
(a) The numbers of papers in each generation of citations to all four key articles			
Generation 2 (papers citing the four key articles)	1082	382	77
Generation 3 (papers citing 77 Central/Important papers from generation 2)	2722	1122	57
Generation 4 (papers citing 46 of the 57 Central/Important papers from generation 3)	541	226	25
Generation 5 (papers citing 18 of the 25 Central/Important papers from generation 4)	170	66	1
Total papers	4515	1796	160

Number of papers included in study	Total citing (%)	Number assessed	Number Central/Important (% of assessed)
(b) The numbers of research articles and reviews across all citing generations of all four key articles passing through the initial filter process based on the number of citation occasions			
Total papers	4515	1796	160 (9)
Research articles	3464	1242	106 (9)
With 3 or more citation occasions	448 (13)	448	89 (20)
With <3 citation occasions	3016 (87)	794	17 (2)
Reviews	1051	554	54 (10)
With 2 or more citation occasions	321 (31)	321	49 (15)
With 1 citation occasion	730 (69)	233	5 (2)

Table 1a shows the distribution of all papers examined across the citing generations of the four key articles. Assessment of generations 2 and 3 were completed for all four key articles, assessment of generation 4 was completed for the streams from three key articles (Kuipers et al. 1997; Burns et al. 1999; Vesa et al. 1995) and generation 5 assessment was completed for the streams from two key articles (Kuipers et al. 1997; Burns et al. 1999). In Table 1a the papers assessed as Central/Important (column 4) in each generation were carried through to the following generation where they became the cited papers. These numbers of cited papers were lower than the numbers of Central/Important papers from the previous generation for generations 4 and 5 due to the assessments for Clark et al. (1994) concluding at generation 3 (Central) and Vesa et al. (1995) at generation 4 (Central).

We examined 3464 citing research articles and 1051 citing reviews. Of these, our groups of assessors assessed 1242 articles and 554 reviews (Table 1b). Therefore 2222 articles and 497 reviews were eliminated from further analysis by the use of our filter mechanism. The groups of assessors found, overall, that the reference was Central for 9 % of assessed citing research articles and Important for 10 % of assessed citing reviews. Thirteen percent (448) of the citing research articles examined had 3 or more citation occasions, all of these were assessed and for 89 (20 %) the reference was considered Central. This compared to the remaining 3016 (87 %) of research articles with less than 3 citation occasions where 794 (26 % of the remainder) were assessed and for 17 (2 % of those assessed) the reference was considered Central. We describe later how we extrapolated from these figures to produce an overall figure for the percentage of citing research articles for which the reference was Central.

For citing reviews, 321 (31 %) had 2 or more citation occasions, all of these were assessed and for 49 (15 %) the reference was considered Important. This compared to the remaining 730 (69 %) reviews which had less than 2 citation occasion, 233 (32 %) of these were assessed and for 5 (2 % of those assessed) the reference was considered Important.

### Variations by key article

Table 2 contains a breakdown of the figures included in Table 1 by key article. Some variation was found across the four key articles. The number of citing research articles throughout the Burns et al. (1999) stream (291) was significantly lower than for the other three key research articles (Kuipers et al. 1997, 975; Vesa et al. 1995, 1192 Clark et al. 1994, 1006). However, across all four key articles the percentage of citing articles (and reviews) found with 3 or more (for reviews 2 or more) citation occasions was reasonably consistent with a mean of 13 % of all citing articles (range 11–15 %) and 31 % of all citing reviews (ranging from 26 to 34 %).

**Table 2:** Numbers of citing papers (research articles and reviews) to all papers in the citation streams that were examined for each of the four key articles

Citing articles	Number citing research articles (% of all)		Number assessed (% of all assessed)		Number (%) classified as Central	
Citation occasions per citing article	Less than 3	3 or more	Less than 3	3 or more	Less than 3	3 or more
(a) All citing research articles; assessed articles; articles where the reference is considered Central with less than three citation occasions and with three or more citation occasions						
Kuipers et al. (1997)	850	125 (13)	226	125 (36)	1 (0.4)	18 (14)
Burns et al. (1999)	250	41 (14)	124	41 (25)	13 (10.5)	20 (49)
Vesa et al. (1995)	1056	136 (11)	247	136 (36)	3 (1.2)	28 (21)
Clark et al. (1994)	860	146 (15)	197	146 (43)	0 (0)	23 (16)
Total	3016	448 (13)	794	448 (36)	17 (2.1)	89 (20)

Citing reviews	Number citing reviews (% of all)		Number assessed (% of all assessed)		Number (%) classified as Important	
Citation occasions per citing review	Less than 2	2 or more	Less than 2	2 or more	Less than 2	2 or more
(b) All citing reviews; assessed reviews; reviews where the reference is considered Important with less than two citation occasions and with two or more citation occasions						
Kuipers et al. (1997)	223	115 (34)	69	115 (63)	3 (4)	22 (19)
Burns et al. (1999)	47	16 (26)	26	16 (38)	1 (4)	2 (13)
Vesa et al. (1995)	250	97 (28)	82	97 (54)	1 (1)	16 (16)
Clark et al. (1994)	210	93 (31)	56	93 (62)	0 (0)	9 (10)
Total	730	321 (31)	233	321 (58)	5 (2)	49 (15)

The findings for the Burns et al. (1999) citation stream also showed a significant difference to the other three citation streams in the percentage of assessed research articles that were Central. This applied whether or not the citing article had three or more citation occasions: 49 % of research articles with three or more citation occasions (compared to an average of 17 % for the other three key articles) and 10.5 % of research articles with less than three citation occasions (compared to an average of 0.6 % for the other three key articles) were assessed as Central. Possible reasons for this variation, which was not apparent for citing reviews, are examined later.

As an additional check on the filter, a complete set of citing papers in one generation of one stream of papers was assessed. We analysed all citing papers in the third generation of the Burns et al. (1999) stream and found very similar results for the percentage of articles with less than 3 citation occasions that were Central as we found for the sample of such papers assessed in the other generations of the Burns stream. Therefore we included the whole of the third generation of citations to Burns et al. (1999) in the continuing analysis.

Using our methodology (i.e. assessing all articles with three or more citing occasions and a sample of those with fewer than three) an average of 36 % of assessed articles (58 % for assessed reviews) had 3 or more (2 or more) citation occasions. The percentages for the Burns et al. (1999) article are lower: 25 % of assessed articles with 3 or more citation occasions and 38 % of assessed reviews with 2 or more citation occasions. This is at least in part because of the inclusion of the whole of the third generation of citing papers.

Finally, we built on finding that the assessment of all the papers in the third generation of Burns et al. (1999) was similar to that for all Burns et al. (1999) generations. This reinforced the view that our assessment of 20 % of the articles with fewer than three citation occasions provided a reasonable reflection of all the articles in a generation with less than three citation occasions. Therefore we extrapolated from the 20 % figure to calculate the total percentage of articles that were Central in the whole citing streams of the four key articles. When calculated as a percentage of the whole body of citing articles examined, adjusting for the percentage of those excluded by our filter, we found 4.4 % of citing articles to be Central. In reviews, we found that the reference was Important for 6.1 % of all those examined.

### Number of citation occasions

**Fig. 1:**
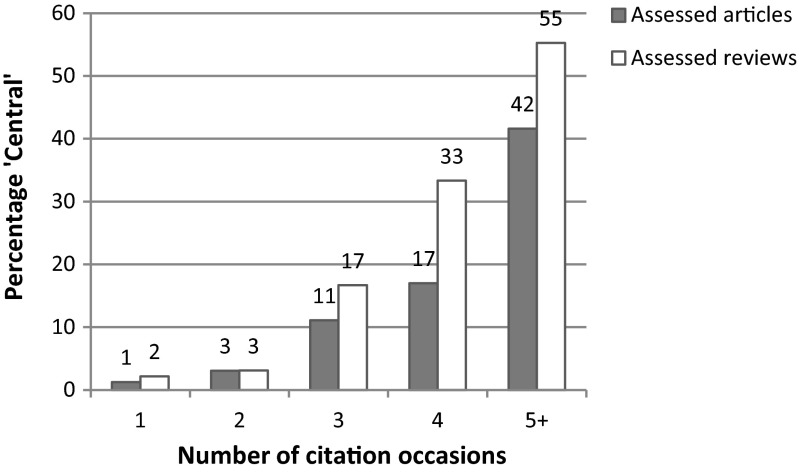
Numbers of citation occasions in assessed papers (research articles and reviews) across all citing generations

An illustration of the number of citation occasions found within the citing articles and reviews and the percentage where the reference was considered to be Central or Important is shown in Fig. 1.

A strong positive correlation was found for both citing articles (*r* = 0.976) and citing reviews (*r* = 0.947). This correlation was more erratic above five citation occasions probably due, at least in part, to the small number of citing articles (113 in total) and reviews (38 in total) that fell into these categories and which we have combined in Fig. 1. There was a noticeable increase in the percentage of articles and reviews where the reference was Central or Important when the number of citation occasions increased from 2 to 3. This reflects the findings from the testing of the prototype (see “Method” section) and is more prominent for reviews than for research articles.

### Agreement between assessors

The level of agreement that was found between assessors for the assessment of research articles and reviews is shown in Table 3.

**Table 3:** The level of agreement found between assessors when assessing all research articles and reviews included in the citation classification procedure

Number of assessors assessing Central/Important	Number of research articles	Percentage of total research articles	Number of reviews	Percentage of total reviews
0	918	74	307	55
1	200	16	161	29
2−	20	2	29	5
2+	52	4	30	5
3	37	3	23	4
4	14	1	5	1

2−, Cases where two assessors considered the reference Central/Important to the citing article/review and neither of the two researchers considered it Central/Important. These papers were not carried through to the next citation generation

2+, Cases where two assessors considered the reference Central/Important to the citing article/review and at least one of the two researchers considered it Central/Important. These papers were carried through to the next citation generation

Using our method of assessment our assessors were in total agreement that the cited paper was not Central for 74 % of citing research articles and not Important for 55 % of citing reviews. There was also total agreement amongst the assessors that the cited paper was Central for 1 % of assessed citing articles and Important for 1 % of assessed citing reviews.

For comparison, a small number of papers were assessed by the experts as part of the pilot study. Out of 44 articles and reviews assessed by both assessors and experts, there was agreement on 26. For the 18 citing papers where assessors and experts did not agree, 16 were considered Central by assessors but not by experts and 2 were considered Central by experts but not by assessors. The general pattern, therefore, reflected the prototype phase where the experts were less likely than the assessors to view a reference as being of importance to the citing paper but there were exceptions.

### The importance of self-citations

A further aspect that we wanted to explore was the numbers of self-citations to the key articles in comparison to non-self-citations and particularly the percentages of self-citations where the reference was considered to be Central and those that led to societal impacts. The findings, included in Table 4, refer to generation 2 for the four key articles, i.e. the citations directly to the four key articles and not to subsequent citing generations.

**Table 4:** Numbers of research articles citing each key article and the percentages that were self-citations: all citing articles; assessed articles; articles considered Central

Number of citing articles	All citing articles		Number assessed		Number Central	
	*n*	Self-cites (%)	*n*	Self-cites (%)	*n*	Self-cites (%)
Kuipers et al. (1997)	135	20 (15)	43	9 (21)	7	5 (71)
Burns et al. (1999)	146	74 (51)	51	27 (53)	16	11 (69)
Vesa et al. (1995)	338	74 (22)	97	32 (33)	18	10 (56)
Clark et al. (1994)	234	21 (9)	79	10 (13)	16	4 (25)
Total	853	189 (22)	270	78 (29)	57	30 (53)

For each key article there is a progressive increase in the percentage of citing articles that were self-cites as we move from all citing articles, through articles assessed and on to articles considered Central

There was considerable variation in the percentages of citing articles that were found to be self-citations for each of the four key articles [9 % for Clark et al. (1994) up to 51 % for Burns et al. (1999)], and this was found to be similarly varied for the percentages assessed [Clark et al. (1994) 13 % and Burns et al. (1999) 53 %] and the percentages considered to be Central. The Burns et al. (1999) and Kuipers et al. (1997) key articles had the highest percentage of Central articles that were self-cites at 69 and 71 % respectively and Clark et al. (1994) had the lowest percentage at 25 %.

### Streams of Central/Important papers

**Fig. 2:**
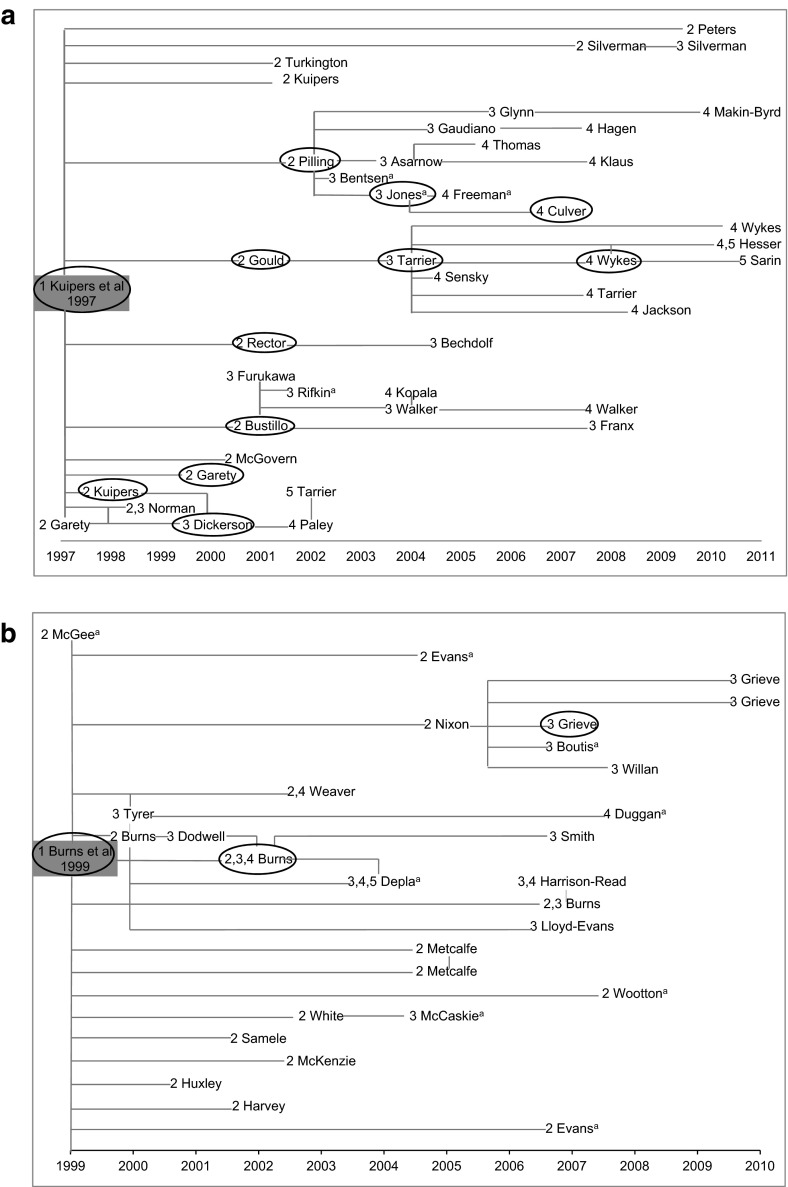

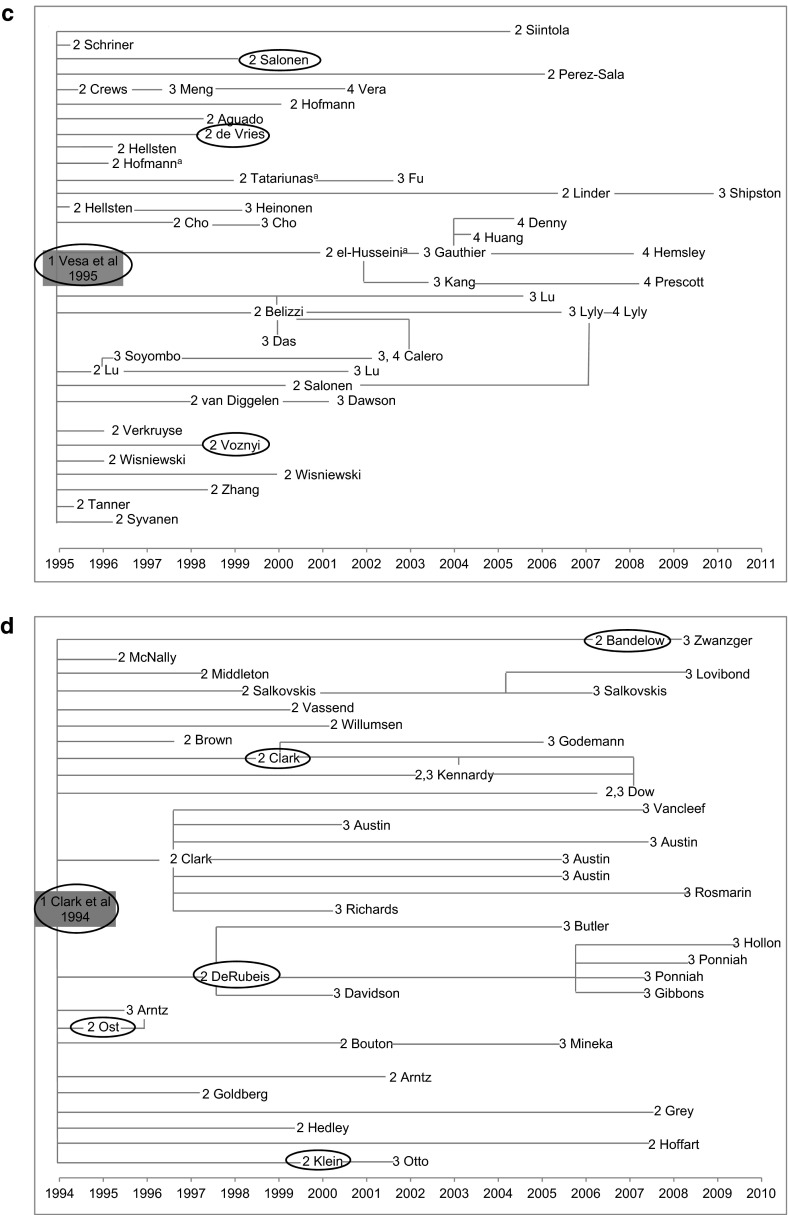
Key articles chosen for study and the citation streams produced using our technique. *Superscript letter a* Papers that would not have been identified using the initial filter based on number of citation occasions. *Oval symbol* Papers cited in societal impacts. *1*, *2*, *3*, *4*, *5* Generations of citing papers for which the cited paper in the previous generation was ‘Central/Important’. **a** Kuipers et al. (1997) and the citing papers in subsequent generations 2–5 for which the reference in the previous generation was Central/Important. **b** Burns et al. (1999) and the citing papers in subsequent generations 2–5 for which the reference in the previous generation was Central/Important. **c** Vesa et al. (1995) and the citing papers in subsequent generations 2–4 for which the reference in the previous generation was Central/Important. **d** Clark et al. (1994) and the citing papers in subsequent generations 2, 3 for which the reference in the previous generation was Central/Important

The citation streams produced from our technique using HACT are illustrated in Fig. 2a–d. The figures illustrate both the time span and the influence of the four key articles found within our study though this influence has quite possibly spread further since. The citing generation where each of the Central articles and Important reviews was identified is included in the figures, pre-fixing the first author’s name (e.g. 2 Gould). Central/Important papers were traced and examined to create up to generation 5 (Central) of the Kuipers et al. (1997) and Burns et al. (1999) streams, generation 4 (Central) for the Vesa et al. (1995) stream and generation 3 (Central) for Clark et al. (1994). The Burns et al. (1999) citation stream as it existed at that time was exhausted within the time frame of our study. We followed up all of the published citations that we were able to access at that time. However more papers citing any of the Central/Important papers in the Burns et al. (1999) stream could have been published or made available later which may have continued the citation stream. For the other three key articles, with more time we would have been able to continue studying more citations and further generations of citations.

### Societal impacts

We explored the societal impacts, for example influence on clinical guidelines or on clinical behaviour as described in case reports, by searching on Web of Science (WoS) for citations initially to the key articles and then to any of the Central/Important papers in the citation streams to the four key articles. In addition we searched on Google for societal impacts to the key articles. The papers that are circled in Fig. 2a–d are those that we identified, within the resources we had available, as the earliest in the citation stream to be included in a societal impact such as a clinical guideline or case report.

For the Kuipers et al. (1997) citation stream, citations in direct and indirect societal impacts were numerous and found to the key article as well as to papers in generations 2–4 (Central). They are described in Table 5.

**Table 5:** Kuipers et al. (1997) citations in societal impacts

Directly citing generation 1 [key article i.e. Kuipers et al. (1997)]: Scottish Intercollegiate Guidelines Network (SIGN) Guideline 30: Psychosocial Interventions in the Management of Schizophrenia (1998) British Columbia Ministry of Health Best Practice document: Core Information Document on Cognitive-Behavioural Therapy (2007)
Citing generation 2 (Central) papers: Two generation 2 (Central) papers were cited in the Royal Australian and NZ College of Psychiatrists clinical practice guidelines for the treatment of schizophrenia and related disorders (2005) American Psychiatric Association (APA) practice guideline for the treatment of patients with schizophrenia (2009) Two generation 2 (Central) papers were cited in a paper providing an overview and describing the scientific base for APA’s 2001 guideline and the relevance for behaviour therapy Italian Guidelines for early intervention in schizophrenia: development and conclusions (2008) Best Practices document from Nebraska Department of Health and Human Services (2005). Influence on training citation demonstrating the evidence-base behind the training requirements in psychotherapy in Canada (2010)
Citing generation 3 (Central) papers: World Federation of Societies of Biological Psychiatry (WFSBP) Guidelines for the Biological Treatment of Bipolar Disorders: Update 2010 on the treatment of acute bipolar depression (2010) Canadian Network for Mood and Anxiety Treatments (CANMAT) guidelines for the management of patients with bipolar disorder: consensus and controversies (2005)
Citing generation 4 (Central) papers numerous generation 4 (Central) papers were cited in Diagnostic guidelines for bipolar disorder: a summary of the International Society for Bipolar Disorders Diagnostic Guidelines workforce (2008) A citation analysing attitudes towards and implementation of NICE guidelines for schizophrenia (2011) uses the generation 4 (Central) paper as essential evidence
Numerous papers in generation 2–4 (Central), were cited in documents providing evidence of impact on practice including papers analysing attitudes towards and implementation of NICE guidelines for schizophrenia and papers illustrating strength of evidence supporting CBT for schizophrenia and showing the levels of use of CBT (2006)

The Burns et al. (1999) key article was directly cited in the Royal Australian and NZ College of Psychiatrists clinical practice guidelines for the treatment of schizophrenia and related disorders (2005), a best practices document from Nebraska Department of Health and Human Services (2005), Assertive Outreach from The Sainsbury Centre for Mental health (2001), a paper linking the World Health Organisation Europe Mental Health Declaration to the evidence supporting it and a paper describing service developments in early intervention in psychosis (2009). A generation 2 (Central) paper was cited in the Evidence-based Guidelines for Treating Bipolar Disorder: Recommendations from the British Association of Psychopharmacology (2003 and 2009 editions). A generation 3 (Central) paper was cited in the International Society for Pharmacoeconomics and Outcomes Research (ISPOR) Good Practices for Quality Improvement of Cost-effectiveness Research Task Force.

The Vesa et al. (1995) key article was cited directly in the European Federation of Neurological Societies (EFNS) task force on molecular diagnosis of neurologic disorders guidelines from the molecular diagnosis of inherited neurologic diseases (2001). Impact on practice was found in numerous case reports citing the key article and some generation 2 (Central) papers in which a breakthrough by Vesa et al. (1995) seemed to contribute to the diagnosis of infantile neuronal ceroid lipofuscinoses (INCL).

Clark et al. (1994) was directly cited, and generation 2 (Central) papers were also cited, in the Royal Australian and NZ College of Psychiatrists Clinical Practice Guidelines for the treatment of panic disorder and agoraphobia (2003) and in the World Federation of Societies of Biological Psychiatry (WFSBP) Guidelines for the Pharmacological Treatment of Anxiety, Obsessive–Compulsive and Post-Traumatic Stress Disorders—first revision (2008). The key article is also cited in a case report in which the use of CBT, supported by Clark et al. (1994), is applied using videoconferencing to a rural area of South Australia (2000). Generation 2 (Central) papers were cited in Canadian Practice Guidelines—Management of Anxiety Disorders (2006) and the Clinical Guidelines for the Treatment of Depressive Disorders. III Psychotherapy from the Canadian Psychiatric Association and Canadian Network for Mood and Anxiety Treatments (CANMAT 2001). Overviews and comments on the empirical base for the American Psychiatric Association Practice Guidelines for Schizophrenia: Scientific Base and relevance for behaviour Therapy (2001) and the American Psychiatric Association practice guidelines for major Depressive Disorder (2001) also included citations to generation 2 (Central) papers.

Having demonstrated the societal impacts that were identified we move on in the Discussion to show how such analysis could be used in impact assessment and be the focus for further developments enabling the approach to be used more widely.

## Discussion

We aimed to explore how far it was legitimate and feasible to assess the impact of health research by using bibliometric methods through a series of generations of citing papers. In order to do this we needed to find a way to identify the citing papers for which the reference was of high importance, then use those papers as the source for the next generation of citing papers, and so on, and finally identify the impact from all the citing generations of papers. We piloted a new approach to classifying citations, designed specifically to facilitate citation analysis across generations of citing papers. Our method included the use of an initial filter based on the number of citation occasions within a citing paper followed by the application of a citation classification template called the HERG Assessment of Citations Template (HACT) based on the importance of a reference to the citing paper. A similar method (Kacmar and Whitfield 2000) involved determination of the influence of a reference to all of its citing papers across one generation of citations. In our pilot study we applied HACT to four chosen key articles across up to five generations of citations, involving a total of 4515 papers, and identified the impacts from all citations to the papers in each generation for which the reference in the previous generation had been of central importance.

In going through a series of generations of citing papers the whole exercise would have rapidly become impractical if all the papers citing the original key articles were then used as the basis for the next generation of citing papers. So, in each generation of papers we identified and focused on what previous research (Kacmar and Whitfield 2000; Prabha 1983; Safer and Tang 2009) suggested would be the very small proportion of citing papers for which the reference was very important. We realised that doing this would be necessary to increase the legitimacy of tracing impact through several generations of citations, but, in practice, it would also enhance the feasibility of the exercise because in each generation the focus on important papers would reduce the number of papers whose citations would form the next generation of citing papers.

The feasibility issues were more acute because the articles that we had chosen to be the key first generation articles for the pilot study (Burns et al. 1999; Clark et al. 1994; Kuipers et al. 1997; Richardson et al. 1988; Vesa et al. 1995) were generally specifically chosen as highly cited articles from streams of work thought likely to show societal impacts. It was possible, therefore, that the subsequent generations of citing papers might also include many citations.

In developing a template for classifying citations we chose to revise the approach originally developed for the prototype template that had been informed by our literature survey (Jones et al. 2012). We found that identifying the number of citation occasions had been the most useful step in comparison to other aspects of the original template. We found that the opinions of the assessors on the use of a reference within the citing paper were too varied, and that in order to trace through to indirect societal impacts we needed to classify the citing papers based on the importance of the reference within the citing paper rather than to categorise the actual role the reference played. This led us to developing and applying HACT, which has similarities to Kacmar and Whitfield’s (2000) assessment of the influence of a reference to the citing paper, though in a different context. The methodology we finally adopted for the pilot, therefore contained two stages, the initial filter based on numbers of citation occasions which would provide us with fast and reproducible verdicts (as suggested by the work of Peritz 1983 and White 2004) on which papers to take through to the second stage which was assessment by applying the HACT. (We had included an additional question in the HACT that provided us with a third middle category of importance of the reference to the citing article. Analysis of this data has not been included here but such an analysis may further inform the qualitative assessment).

We explored how far the application of our method might produce results that were comparable with previous studies based on categorisations of citations. We had found, by extrapolation to account for papers excluded by our initial filter, that the reference was Central to 4.4 % of articles and Important to 6.1 % of reviews in the whole body of citing papers examined. These figures fall somewhere between Hanney et al.’s (2005) findings from categorisation of one generation of citations that for 8 % of cases the reference was of considerable importance to the citing paper and for 1 % it was essential. It is also in agreement with other findings that the reference was highly important for only a small proportion of citations (Prabha 1983; Safer and Tang 2009). Kacmar and Whitfield’s (2000) findings, using different criteria, reported that 9 % of *AcademyofManagementReview* papers and 6 % of *AcademyofManagementJournal* papers were important.

In the pilot study we found that the reference was Central to the citing article 10 times more frequently if it was cited on three or more occasions compared to those articles containing less than three citation occasions (Table 1). For references in reviews, the difference was only slightly lower with 7.5 times the proportion considered Important if there were two or more citation occasions. This finding supports our use of the initial filter as a first step in our assessment method for both research articles and reviews. These findings are also in agreement with previous research findings (McCain and Turner 1989; Peritz 1983; Safer and Tang 2009; Tang and Safer 2008). The correlation between the number of citation occasions and the degree of connection between the cited and citing papers has been discussed for many years. Voos and Dagaev discussed it in 1976 (Voos and Dagaev 1976) and more recently Zhu et al. (2015) found that the number of times a reference was mentioned in the body of the paper was the best of those features examined at predicting those references identified by the author as most important to their paper. Similarly Hou et al. (2011) had considered counting citations in texts to be a more accurate means of assessing the scientific contribution of such references than counting citations in the reference list.

Nevertheless, our findings also showed that a very small proportion of citing articles with fewer than three citing occasions were also classified as ones for which the reference was Central. Furthermore, because there were very many more citing articles with fewer than three citation occasions to a reference than ones with three or more citation occasions it was clear that the number of Central articles that would be missed by the application of the filter would not be negligible. We should, however, note the differences found across the citation streams of the four key articles. The citation stream for Burns et al. (1999) contains 13 out of the total of 17 articles with less than three citation occasions where the reference was Central. A potential reason for this is discussed later but here we consider all four citation streams together. With a potentially significant number of Central articles being missed, the usefulness of the filter would clearly depend on the purposes for which HACT was being applied. In particular, if it was to provide possible additional ways to identify the societal impacts from research, which was the starting point for our project, then the use of the filter could make an important contribution to the feasibility of doing so by focusing attention on the articles that are ten times more likely to be viewed as Central. And this part of the process could also be speeded up through automation, a step considered to be very beneficial by White (2004). If the intention of using HACT was to provide a fully comprehensive analysis then the use of the filter would become more problematic and further investigation about how to deal with the Central articles that would be missed out would clearly be of benefit.

One aspect of qualitative citation assessment that has been much debated and that we chose to address was the question of self-cites; should they be included or not? When a quantitative citation assessment is conducted it is generally considered prudent to omit all self-cites as they can lead to distortion of the value of the assessment (Harzing 2010). However the value of a self-cite could be argued to potentially be of greater importance to the development of a research theme than a non-self-cite (Prabha 1983; Safer and Tang 2009; Snyder and Bonzi 1989; Tang and Safer 2008). According to Hartley (2012) the reasons for self-cites were varied and therefore he argued that it was not correct to consider them all as purely self-enhancing. Although Hartley was discussing self-cites in relation to journal impact factor his thoughts relate to the use of self-cites by an author and how these can vary within a paper and therefore are also informative in this context. He argued that it is the role of the self-citation within the paper that is important in assessing the contribution made by the reference. Kacmar and Whitfield (2000) also examined self-cites and found considerable variation in percentage from one paper to another and that most of those with the larger numbers of self-cites also had greater than average levels of citing papers where the reference was important. They discussed that some researchers appeared to be focusing their research in one particular area which resulted in a greater number of self-cites in their publications. We examined the self-citations in generation 2, i.e. in citations to just the key articles, due to the difficulties with the definition of a self-cite in subsequent citation generations. We found some variation in the percentage of citations to the key articles that were self-cites but we found that for all four of the key articles, a greater proportion of self-citing articles were Central than non-self-cites, though the numbers were quite small. Additionally, for three of the four key articles the majority of Central articles were self-cites. These findings agree with those of Tang and Safer (2008) and lead to the suggestion that self-cites may have a greater level of importance than non-self-cites when tracing the influence of research and therefore supports their inclusion in a qualitative evaluation.

When considering our findings for the citation streams of the four key articles, we found many similarities including the percentage of citing articles with three or more citation occasions, the percentage of reviews with two or more citation occasions and the percentages of reviews where the cited paper was considered Important whether it was cited more than twice or not. We also found some differences and this was particularly noticeable for the Burns et al. (1999) stream in comparison to the citation streams for the other three key articles. The cited paper was Central for a much higher percentage of the citing articles in the Burns et al. (1999) stream and citations to the Burns et al. (1999) key article included a much higher percentage of self-cites than the other three articles. Both of these findings applied whether or not the cited paper was cited on three or more citation occasions. Nevertheless, the percentage of Central articles that were self-cites was as high for the Kuipers et al. (1997) key article as for Burns et al. (1999) (Table 4). Previous work (Hanney et al. 2005) has shown that the authors of the self-citing articles would often write in an authoritative way about how they were building on their own previous work, even if they did not find it necessary to cite it on three or more occasions. This is also supported by Safer and Tang’s findings (2009) that not only were self-citations more important than non-self-cites and that this was independent of the number of citation occasions within the text but also that authors judged their prior research as very important for their research article even if it was cited just once in the introduction. This issue, together with our findings, may indeed lead to a need for self-cites to be considered separately to non-self-cites.

The citation streams created by use of our selective qualitative citation analysis method illustrate the influence of the key articles across subsequent citing papers. The selective nature of our method could be likened to Kostoff’s ‘radioactive tracer’ (1998) allowing us to trace influence across up to five citing generations and on to the indirect societal impacts of health research. Figure 2a–d show the citing papers where the reference was considered Central/Important from the key article across up to five citing generations. These citation streams illustrate the sometimes linear pathway of citation generations and also more complex interactions with many papers being included in more than one citing generation. The length of time passed between cited and Central/Important citing paper varies widely. Some generation 2 (Central) papers were published in the same year as the key article, maybe indicating that those papers were based on the same data source as the key article, and others were published some 13 years later for Kuipers et al. (1997) and 14 years later for Clark et al. (1994). The Vesa et al. (1995) citation stream contained more Central/Important citations within the first 3 years after publication of the key article in comparison to the other three key articles. This may reflect differences in research areas as Vesa et al.’s (1995) work is closer to basic neuroscience whilst the other three key articles are closer to the social psychiatry end of the mental health research spectrum.

Over the time that the research project was conducted, we assessed citations across up to five generations for the Kuipers et al. (1997) and Burns et al. (1999) key articles, and as far as four generations for the Vesa et al. (1995) key article and three generations for Clark et al. (1994). We then used all of the Central/Important papers in all generations of citations to each key article to try to identify the direct and indirect societal impacts. This entailed us going further than in previous studies, including those using the Payback framework (Hanney et al. 2013), where one generation of citing papers was examined to see how far it included items such as clinical guidelines that could be counted as societal impacts from the research. In our current analysis we identified numerous examples of societal impacts by searching Web of Science for citations to any Central/Important paper and additionally by searching Google for citations to the key articles. A major strength of the citation analysis was in the number of clinical guideline documents that we identified in these citation searches. These citations, that sometimes occurred generations later, illustrated the international importance of the work described in the key articles, in conjunction with other published research. On the databases accessed it was striking how much of the impact was first associated with later generations of papers in the citation streams, impact that is usually not easily identified and may be not known to the original authors. Sometimes more than one of the papers included in the citation streams was cited in a document identified as a societal impact therefore in Fig. 2a–d we have marked just the earliest Central/Important paper that was cited. Sibbald et al. (2015) described the importance of including grey literature in a study of research impact. As the transition from research to clinical impact is complex perhaps the range of information sources used to identify the societal impacts add to our understanding of the complexity involved, especially in the way in which we have presented it in our series of figures that highlight the timelines over which the impacts arose.

Drawing all this together we believe the approach we have developed could add further dimensions to the growing range of approaches that exist to demonstrate the societal impacts from research (Banzi et al. 2011; Bornmann 2013; Buxton and Hanney 1996; Guthrie et al. 2013; HEFCE 2015; Milat et al. 2015). The existing approaches vary in nature and scope, and have been developed to meet the increasing demand on researchers and research funders to demonstrate the societal impacts of the research they fund. There is increasing interest in collecting data on the impact made by health research on clinical guidelines (Turner et al. 2015). Furthermore, an impact on guidelines was reported to be one of the most frequently claimed impacts in the case studies produced in the recent REF2014 by health and life science research departments in UK universities to demonstrate how they had benefitted wider society (HEFCE 2015).

Case studies conducted to identify the impact of specific pieces or streams from research can draw on a range of data sources (Hanney et al. 2013). Because our approach works through a series of generations the approach might illustrate the level of influence that the bodies of work have had in a range of ways that other impact assessment approaches might be less able to identify. For example, our method could provide an additional tool for assessing the impact of knowledge production on other policy-relevant research and on clinical policies such as in clinical guidelines. And at the international level our approach would possibly take things further than impact assessment analyses that rely on the knowledge of the researchers and/or their local peers. The potential relevance of our approach was illustrated in the recent REF2014 exercise in the UK. The Kuipers et al. (1997) key article formed an important part of an impact case study conducted as part of submission from King’s College London (Higher Education Funding Council 2015). The type of analysis facilitated by our approach could potentially have contributed further data for the analysis.

Furthermore, assessments of research impact can also be conducted in order to understand more about the processes through which the societal impacts arise (Wooding et al. 2014a, b). It is widely recognised that pathways to impact might be long, and many streams of work can make a contribution to the societal impacts that arise. Here, too, conducting case studies informed by our approach would feed into discussions about the pathways through which impacts can be achieved. Applying our approach opens up a new way of analysing impact through a series of generations of papers which might help shed light on processes that had previously been assumed to be happening but which had not been properly mapped. This complements other recent work that provides greater understanding of how to analyse the elapsed time (more commonly called time lags) between early research and its eventual impact on health policies and practice (Hanney et al. 2015).

### Limitations and next steps

The approach developed here is very resource intensive as is traditional peer review. We realised it would be impossible to make extensive use of experts on such a large-scale enterprise. We therefore relied considerably on non-experts, mostly post-graduate students in other fields, to assess the importance of citations. We compared the majority verdict of our group of non-expert assessors with the findings of the experts who we did involve in the project in a highly selective way. This revealed that the post-graduate assessors tended to be less discriminating and had a lower threshold at which they consider a reference to be important when compared to expert judgement. This has potentially resulted in an over-estimation of the numbers of Central/Important papers using our method relative to expert view. It is, however, extremely unlikely that even a group of experts would completely agree that a paper was Central/Important. We found in the pilot, as we had previously in our test of the prototype template, that perfect agreement by all experts on the ‘Centrality/Importance’ of a reference was not achieved. Hanney et al. (2005) had also found that complete agreement between assessors was rare. Therefore, employing a majority verdict approach by groups of four postgraduate assessors might have provided a reasonable compromise between use of resources and ‘accuracy’ of the findings and we found agreement between at least three out of four assessors for 90 % of citing articles and for 84 % of citing reviews.

While tracing the influence of key research articles over many generations of citations by selectively identifying those citations where the reference is Central/Important is an interesting exploration of the progress from research to societal impacts, the level of influence that the key article may have had on the indirect societal impact is speculative unless examined and assessed carefully. Further studies using HACT, probably supplemented by the use of the filter, could contribute further understanding of the influence of a citation across many subsequent citation generations, which has been an issue of interest at least since Rousseau’s pioneering 1987 article (1987) although that considered academic impact as opposed to societal impact. An examination of more key articles by this method would expand our understanding of the variations in influential citation streams for different key articles and the spread of influence of key pieces of health research on the societal impacts. Furthermore, despite the progress in tracing societal impacts, and the ability to conduct web-based searches, access to the grey literature is often challenging and time consuming as Sibbald et al. (2015) note.

A range of questions arose about what would be the most appropriate starting point for the analysis. Further work on identifying which paper should be selected as the appropriate starting point and whether a body of work should be used rather than a single key article would help to present a more accurate picture of the development of the societal impacts of health research.

Further research that could help refine the assessment procedure described here could include an investigation of automated procedures that could usefully be applied at some steps of the procedure to increase the efficiency of the process. Zhu et al. (2015) suggested that the counting of citation occasions would be one of the easiest additional aspects of citation analysis to automate and Teufel et al. (2006) have previously developed an automated procedure that can identify citation occasions within a paper. More analysis of the role or importance of self-citations relative to non-self-citations could further inform the use of objective measures in a qualitative citation analysis. Tang and Safer (2008) had found that the relationship between objective measures such as citation occasions and the importance of a reference were weaker for self-citations.

We have shown that we can use our new approach to contribute towards assessing the societal impacts of research, and help understand the processes involved in achieving research impact. However, on a case by case basis, it might be worth considering when this new approach contributes sufficient additional data to that provided from other sources, to justify the resources that would be used in conducting the analysis. We could possibly further inform this by a fuller analysis of both data already gathered from authors and experts in the course of our project, and new data emerging for the REF2014 exercise in the UK (Higher Education Funding Council 2015) which included assessment of the societal impacts of research, for example the case study involving Kuipers et al. (1997). Despite the extensive nature of our analysis we are aware there were further steps we could have taken had resources allowed, including exploring the impacts of any previous generation of (cited) clinical guidelines and case reports on the next generation of (citing) clinical guidelines and case reports and exploring the importance of a citation within a clinical guideline or case study. The new method described here should perhaps not be viewed as a comprehensive method of identifying the societal impacts of a body of research. It has, however, identified a number of important impacts and pathways to impact on clinical practice and policy made by some of the chosen key articles.

## Conclusions

Following a systematic review of the literature on the meaning and use of citations (Jones et al. 2012), we developed a simple template to qualitatively assess a reference’s importance to the citing paper in which it appears. We applied the template, called the HACT, in a pilot study to qualitatively assess the citations to four chosen key research articles from the area of psychiatry/neuroscience and then to similarly assess citations to those papers for which the key article was Central/Important. To increase the legitimacy of the study we applied an initial filter based on the number of citation occasions so that we did not classify all the citing papers. We identified a suitable cut-off point of three or more citation occasions.

We have found that the reference was Central for 4.4 % of citing articles overall and it was Central ten times more frequently for those articles containing three or more citation occasions. We found more self-cites to be Central than non-self-cites. We traced through up to five generations of citations in order to create a citation stream of influence for each of the key articles. We then conducted a citation search on the Web of Science of all the papers in each generation, as well as a citation search of the key articles on Google, in order to identify the direct and indirect societal impacts such as a citation on a clinical guideline. We identified societal impacts in the citation streams including citations in international clinical guidelines. We believe that we have shown that this is an approach that should be further explored given the increasing interest in identifying the societal impacts of research as it potentially provides an additional qualitative method of exploring the influence that a piece of biomedical research has had and in illuminating the processes involved in the translation of the research findings and the eventual impact on clinical practice.

## Electronic supplementary material

Below is the link to the electronic supplementary material.

Online resource 1: Prototype templates and pilot templates [HERG Assessment of Citations Template (HACT)] for original research articles and reviews (DOCX 46 kb)

Online resource 2: Panel of experts providing guidance throughout the project. Analysis of the application of the prototype templates (DOCX 20 kb)
