# Supplementary material for: Tracing the indirect societal impacts of biomedical research: development and piloting of a technique based on citations
Source: Scientometrics. 2016 Mar 8;107:975–1003. doi: 10.1007/s11192-016-1895-4 (PMC4869749; doi:10.1007/s11192-016-1895-4)
Supplement: Supplementary file 1 — Online resource 1: Prototype templates and pilot templates [HERG Assessment of Citations Template (HACT)] for original research articles and reviews (DOCX 46 kb) [file 11192_2016_1895_MOESM1_ESM.docx]

Tracing the indirect societal impacts of biomedical research: development and piloting of a technique based on citations

*Scientometrics*

Jones, Teresa H* and Hanney, Steve

Health Economics Research Group, Brunel University London, Uxbridge, Middlesex UB8 3PH, UK

*Corresponding author
*e-mail* [teresa.jones@brunel.ac.uk](mailto:teresa.jones@brunel.ac.uk)
*telephone*: +44 (0)1895 267558
*Fax*: +44 (0)1895 269708

Online resource 1: Templates

Prototype template for published original research articles

Prototype template for reviews and discussion articles

HERG Assessment of Citations Template (HACT)

- Pilot template for original research articles
- Pilot template for reviews and discussion articles

Prototype template for published original research articles

**Published original research articles. Datasheet: Categorising citations**

Please complete your initials and the date before starting the assessment. You will be answering questions about the citing article.

**Cited article** ID: A

**Authors:**

**Title:**

**Journal:**

**Vol:** **Pages:**  **Year**:

Initials of reviewer………………………

Date of assessment……………………

Pdf/paper/other electronic (Please circle)

**Citing article** ID: B

**Authors:**

**Title:**

**Journal:**

**Vol**: **Pages**:  **Year**:

What style is the bibliography?

Harvard (Author names)….

Vancouver (Numbers)……. Number…….

## 1. Individual citation occasion details

Please consider each citation occasion separately. Locate each highlighted citation occasion in the copy of the citing article and make an assessment using the guidance provided in the instruction sheet. **Note/Review only** is the *default* position and should be ticked unless there is explicit evidence of greater involvement of the citation occasion.

| Citation occasion | Page number | Location  Intro/Method/Results/Discuss | First author named in text | Note/  Review only | Fuller use |
| --- | --- | --- | --- | --- | --- |
| 1^st^ |  |     |  |  |  |
| 2^nd^ |  |     |  |  |  |
| 3^rd^ |  |     |  |  |  |
| 4^th^ |  |     |  |  |  |

**If either of the following conditions apply then please proceed on to Question 2 below:**

- **there are three or more citation occasions marked ‘Note/Review only’**
- **any of the citation occasions is marked ‘Fuller use’.**

**2. Whole article details**

Please consider the citing article as a whole. Read around the citation occasions as much as is considered necessary, first at sentence level, progressing to whole paragraph level and so on until an assessment can be made.

1. Please tick the boxes, *if appropriate,* according to the definitions and examples provided in the instruction sheet and your own judgement. **You may tick more than one box**.

| **Apply:** The citing article applies or replicates methods, technologies, theories, scales etc found in  the cited article in a similar way as described in the cited article**.** |  |
| --- | --- |
| **Develop:** The citing article is further developing (partially or totally) a concept or method  previously described in the cited article either by modification or different application. |  |
| **Refute:** The citing article *either* claims that the cited article is incorrect *or* disputes (partially or  totally) the cited article but is unable to come to a firm conclusion. |  |

1. Please tick whichever box is most applicable according to the definitions provided and your own judgement BUT the *default* position is to tick ‘**Not highly important’**.

| **Highly important**: The cited paper is considered to be necessary for the research described in  the citing paper to have taken place i.e. BUT FOR the cited paper the citing paper could not have  been written. The key conclusions significantly depend on applying, replicating, developing or  refuting the cited article. |  |
| --- | --- |
| **Not highly important:** *(Default)* The cited paper is NOT considered to be necessary for the  research described in the citing paper to have taken place. |  |

Notes: *Please add any comments that you wish to record here.*

**Published original research articles. Instruction Sheet: Categorising citations**

We are tracing the impact of biomedical research as part of an MRC-funded project and are collecting information on the relationships between cited and citing articles. You have been provided with one datasheet per citing article and a copy of the citing article. The following notes are provided for your guidance but if you need further clarification on completing this datasheet please contact Teri Jones on 01895 265492 or e-mail [Teresa.Jones@brunel.ac.uk](mailto:Teresa.Jones@brunel.ac.uk)

**Use this datasheet to assess citations within published articles or notes describing original biomedical research.** Each citation occasion of the cited article has been highlighted in the copy of the paper provided and some details relating to each citation occasion have been completed in the table.

## 1. Individual citation occasion details

Please consider each citation occasion separately. Locate each highlighted citation occasion in the copy of the citing article and make an assessment using this guidance.

**Note/Review only:** This category is the *default* position and should be ticked unless there is explicit evidence of greater involvement of this citation occasion. This category covers the citing article referring to the cited article as part of the relevant literature but either serving no explicit role in the discussion or analysis (note) or being compared to other relevant literature (review). This judgement could include: just a reference number in the Vancouver referencing style e.g. [34] or [32-36] or the name of the paper for the Harvard style e.g. [Smith et. al. 2004] or [Smith et al, 2004, Brown et al, 2003 and Morgan et al, 2005].

**Fuller use**: Any explicit evidence of greater involvement of the citation at this citation occasion over and above Note/Review only e.g. explicit evidence in a single sentence of a greater level of involvement or importance; extended discussion across more than one sentence; inclusion of a quotation from the cited paper.

**2. Whole article details**

Please consider the citing article as a whole. Read around the citation occasions as much as is considered necessary, first at sentence level, progressing to whole paragraph level and so on until an assessment can be made.

A. Please tick the boxes, *if appropriate,* according to these definitions and examples and your own judgement. **You may tick more than one box**.

**Apply:** The citing article applies or replicates methods, technologies, theories, scales etc found in the cited article in a similar way as described in the cited article**.**

**Develop:** The citing article is further developing (partially or totally) a concept or method previously described in the cited article either by modification or different application.

*Example*. A concept or method in the cited paper is described in the Introduction/Method AND reference is made in the Discussion/Conclusion to how this concept or method has worked or can be compared.

**Refute:** The citing article *either* claims that the cited article is incorrect *or* disputes (partially or totally) the cited article but is unable to come to a firm conclusion.

B. Please tick whichever box is most applicable according to the definitions provided and your own judgement BUT the *default* position is to tick the ‘**Not highly important’** box.

**Highly important**: The cited paper is considered to be necessary for the research described in

the citing paper to have taken place i.e. BUT FOR the cited paper the citing paper could not have

been written. The key conclusions significantly depend on applying, replicating, developing or

refuting the cited article.

**Not highly important:** *(Default)* The cited paper is NOT considered to be necessary for the research described in the citing paper to have taken place.

Prototype template for reviews and discussion articles
**Reviews and discussion articles. Datasheet: Categorising citations.**

Please complete your initials and the date before starting the assessment. You will be answering questions about the citing article.

**Cited article** ID: A

**Authors:**

**Title:**

**Journal:**

**Vol:** **Pages:**  **Year**:

Initials of reviewer………………………

Date of assessment……………………

Pdf/paper/other electronic (Please circle)

**Citing article** ID: B

**Authors:**

**Title:**

**Journal:**

**Vol**: **Pages**:  **Year**:

What style is the bibliography?

Harvard (Author names)….

Vancouver (Numbers)……. Number…….

Systematic Review (Please circle)……Yes/No

## 1. Individual citation occasion details

Please consider each citation occasion separately. Locate each highlighted citation occasion in the copy of the citing article and make an assessment using the guidance provided in the instruction sheet. **Note/Review only** is the *default* position and should be ticked unless there is explicit evidence of greater involvement of the citation occasion.

| Citation occasion | Page number | Location  Intro/Body of article/Discuss | First author named in text | Note/  Review only | Fuller use |
| --- | --- | --- | --- | --- | --- |
| 1^st^ |  |    |  |  |  |
| 2^nd^ |  |    |  |  |  |
| 3^rd^ |  |    |  |  |  |
| 4^th^ |  |    |  |  |  |

**If either of the following conditions apply then please proceed on to Question 2 below**

- **there are three or more citation occasions marked ‘Note/Review only’**
- **any of the citation occasions is marked ‘Fuller use’.**

**2. Whole article details**

Please consider the citing article as a whole. Read around the citation occasions as much as is considered necessary, first at sentence level, progressing to whole paragraph level and so on until an assessment can be made.

1. Please tick the boxes, *if appropriate,* according to the definitions provided and your own judgement. **You may tick more than one box**.

| **Support:** The citing article is supporting (partially or totally) a concept or method or results described in the cited article. |  |
| --- | --- |
| **Refute:** The citing article either claims that the cited article is incorrect or disputes (partially or totally) the cited article but is unable to come to a firm conclusion. |  |

1. Please tick whichever box is most applicable according to the definitions provided and your own judgement BUT the *default* position is to tick ‘**Not highly important’**.

| **Highly important**: The cited paper is considered to form a necessary part of the discussion/conclusions reached for the topic under discussion/review. |  |
| --- | --- |
| **Not highly important:** *(Default)* The cited paper is NOT considered to form a necessary part of  the discussion/conclusions reached for the topic under discussion/review. |  |

| Notes: *Please add any comments that you wish to record here.* |
| --- |

**Reviews and discussion articles. Instruction Sheet: Categorising citations.**

We are tracing the impact of biomedical research as part of an MRC-funded project and are collecting information on the relationships between cited and citing articles. You have been provided with one datasheet per citing article and a copy of the citing article. The following notes are provided for your guidance but if you need further clarification on completing this datasheet please contact Teri Jones on 01895 265492 or e-mail [Teresa.Jones@brunel.ac.uk](mailto:Teresa.Jones@brunel.ac.uk)

**Use this datasheet to assess citations within published reviews or discussion papers.**

**Reviews**. If you consider the citing article that you are studying to be a review only, then please complete this datasheet, marking whether or not it is a systematic review. If, however, the article contains a literature review as part of a larger piece of work that is not a review then it should be assessed using the alternative form ‘*Published original research articles. Datasheet: Categorising citations’.*.

**Discussion** **papers**. This type of paper may not follow the usual structure of a scientific paper (Intro/Method/Results/Discuss). It should be assessed using this datasheet.

Please complete your initials and date before starting the assessment. You will be answering questions about the citing article.

## 1. Individual citation occasion details

Please consider each citation occasion separately. Locate each highlighted citation occasion in the copy of the citing article and make an assessment using this guidance.

**Note/Review only:** This category is the *default* position and should be ticked unless there is explicit evidence of greater involvement of this citation occasion. This category covers the citing article referring to the cited article as part of the relevant literature but either serving no explicit role in the discussion or analysis (note) or being compared to other relevant literature (review). This judgement could include: just a reference number in the Vancouver referencing style e.g. [34] or [32-36] or the name of the paper for the Harvard style e.g. [Smith et. al. 2004] or [Smith et al, 2004, Brown et al, 2003 and Morgan et al, 2005].

**Fuller use**: Any explicit evidence of greater involvement of the citation at this citation occasion over and above Note/Review only e.g. explicit evidence in a single sentence of a greater level of involvement or importance; extended discussion across more than one sentence; inclusion of a quotation from the cited paper.

**2. Whole article details**

Please consider the citing article as a whole. Read around the citation occasions as much as is considered necessary, first at sentence level, progressing to whole paragraph level and so on until an assessment can be made.

1. Please tick the boxes, *if appropriate,* according to these definitions and your own judgement.

**You may tick more than one box**.

**Support:** The *citing* article is supporting (partially or totally) a concept or method or results described in the cited article.

**Refute:** The *citing* article either claims that the cited article is incorrect or disputes (partially or totally) the cited article but is unable to come to a firm conclusion

B. Please tick whichever box is most applicable according to the definitions provided and your own judgement BUT the *default* position is to tick the ‘**Not highly important’** box.

**Highly important**: The cited paper is considered to form a necessary part of the discussion/conclusions reached for the topic under discussion/review

**Not highly important:** *(Default)* The cited paper is NOT considered to form a necessary part of the discussion/conclusions reached for the topic under discussion/review

HERG Assessment of Citations Template (HACT): Pilot template for original research articles

*ORIGINAL RESEARCH ARTICLE*

*Question 1*: Is the cited article CENTRAL to the message of this paper? The DEFAULT position is NO.

*Guidance for Q1:*

Tick yes if the KEY CONCLUSIONS of this paper as a whole, could not have been reached without one or more of the following?

- - By applying a novel theory, method, scale or technology, etc set out in the cited article.
  - By supporting or developing, either by modification or different application, a concept or method set out in the cited article.
  - By refuting a concept or method from the cited article

Yes – end of assessment of this paper

No – please continue onto Question 2

*If your answer to Question 1 was NO then please answer Q2.*

*Question 2*: Is the cited article used to make a SIGNIFICANT CONTRIBUTION to the argument in at least one part of this paper? The DEFAULT position is NO.

*Guidance for Q2:*

Tick yes if this paper:

- - Describes some aspect of the method, findings or conclusions of the cited article in detail in at least one full sentence and not simply “as Smith has shown” OR “see Smith, 2008”.
    *Example 1*. “The current finding is also in line with the cognitive behavioural model of OCD which proposes that negative appraisals lead OCD patients to require unusually stringent criteria for deciding that an action has been appropriately completed (Salkovskis, 1999).”
    *Example 2.* “The findings of Wroe and Salkovskis (2000) suggest that, in such situations, the decision not to act to prevent possible harm is viewed by individuals with OCD as being equivalent to causing the harm in the first place.”
  - Includes a quotation from the cited article

Yes

No

*END OF ASSESSMENT*

HERG Assessment of Citations Template (HACT): Pilot template for reviews and discussion articles

*REVIEW OR DISCUSSION ARTICLE*

*Question 1*: Is the cited article IMPORTANT to a key message from this review/discussion paper? Please consider this paper as a whole and that the DEFAULT position is NO.

*Guidance for Q1:*

Tick yes if the cited article is used to help reach or sustain a KEY TAKE-HOME MESSAGE or CONCLUSION of this review/discussion paper.

i.e. There is one or more citation occasion which describes the cited paper in some detail (likely to be at least one full sentence)

**AND** that or another citation occasion occurs at a point in the text where a key conclusion or take-home message from the review is being developed or discussed.

Yes

No

END OF ASSESSMENT
