# Supplementary material for: Tracing the indirect societal impacts of biomedical research: development and piloting of a technique based on citations
Source: Scientometrics. 2016 Mar 8;107:975–1003. doi: 10.1007/s11192-016-1895-4 (PMC4869749; doi:10.1007/s11192-016-1895-4)
Supplement: Supplementary file 2 — Online resource 2: Panel of experts providing guidance throughout the project. Analysis of the application of the prototype templates (DOCX 20 kb) [file 11192_2016_1895_MOESM2_ESM.docx]

Tracing the indirect societal impacts of biomedical research: development and piloting of a technique based on citations

*Scientometrics*

Jones, Teresa H* and Hanney, Steve

Health Economics Research Group, Brunel University London, Uxbridge, Middlesex UB8 3PH, UK

*Corresponding author
*e-mail* [teresa.jones@brunel.ac.uk](mailto:teresa.jones@brunel.ac.uk)
*telephone*: +44 (0)1895 267558
*Fax*: +44 (0)1895 269708

Online resource 2:

Panel of experts providing guidance throughout the project

Analysis of the application of the prototype templates

**Panel of experts providing guidance throughout the project**

- Professor Tom Burns, Chair of Social Psychiatry, University of Oxford
- Dr Jon Cooper, (now Professor of) Experimental Neuropathology, Institute of Psychiatry, King’s College London
- Dr Claire Donovan, Reader in Assessing Research Impact, Brunel University London
- Dr Chris Henshall: Honorary Professor, Brunel University London; Health, Research & Innovation Policy Consultant.
- Professor Peter Jones, Head of Community Psychiatry, University of Cambridge.
- Dr Amy Pooler, Post-doctoral Research Fellow specialising in research into Alzheimer’s Disease, Institute of Psychiatry, King’s College London

**Analysis of the application of the prototype template.**

Of the 96 papers included in the assessment process 75 were research articles and 21 were reviews/discussion papers. The chief criterion of the analysis was considered to be an assessment of the reference as highly important or not highly important to the citing paper.

Table 1 contains details of the analysis of the findings for assessed research articles. For 70 of the 75 assessed articles all experts and a majority of reviewers agreed that the cited article was not highly important to the citing article. For the five assessed articles where at least one expert considered the reference to be highly important, there were no occasions where all four experts agreed. Out of the 11 reviewers, between two and eight thought that the reference was highly important to these five citing articles. An examination of the characteristics of these assessed papers showed a correlation between numbers of citation occasions and high importance of the reference. All five citing articles where at least one expert thought that the reference was highly important cited the reference on at least three occasions. In addition there were nine citing papers with three or more citation occasions where no experts but at least two reviewers considered the cited reference to be highly important.

**Table 1.** Results from the assessment of 75 research articles

| *Combinations of number of assessors classifying the reference as being highly important to the citing paper* | | *Number of examples of each combination of assessment by subject experts and reviewers* | |
| --- | --- | --- | --- |
| *Subject experts n=4* | *Reviewers n=11* | *Total number of each combination* | *Examples with =/>3 citation occasions in the citing paper* |
| 3 | 8 | 1 | 1 |
| 3 | 6 | 1 | 1 |
| 2 | 5 | 1 | 1 |
| 2 | 2 | 1 | 1 |
| 1 | 8 | 1 | 1 |
| 0 | 5 | 1 | 1 |
| 0 | 4 | 4 | 3 |
| 0 | 3 | 4 | 2 |
| 0 | 2 | 9 | 3 |
| 0 | 1 | 19 | 0 |
| 0 | 0 | 33 | 0 |
| - | - | **Total = 75** | **Total = 14** |

The findings from the assessment of reviews can be found in Table 2. For one citing review out of the 21 assessed, the reference was considered highly important by an expert, and that was by just one of the four experts. The paper cited in this review was also considered highly important to the review by the majority, six out of eleven, of reviewers. This review contained three or more citation occasions. There were 12 other reviews, four with three or more citation occasions, where a minority of the reviewers (ranging from one to four), thought that the reference was highly important to the review.

**Table 2.** Results from the assessment of 21 review articles

| *Combinations of number of assessors classifying the reference as being highly important to the citing paper* | | *Number of examples of each combination of assessment by experts and reviewers* | | |
| --- | --- | --- | --- | --- |
| *Subject experts, n=4* | *Reviewers, n=11* | *Assessed n=21* | *"=/>2 citation occasions"* | *"=/>3 citation occasions"* |
| 1 | 6 | 1 | 1 | 1 |
| 0 | 4 | 1 | 1 | 1 |
| 0 | 3 | 1 | 1 | 1 |
| 0 | 2 | 4 | 3 | 2 |
| 0 | 1 | 6 | 2 | 0 |
| 0 | 0 | 8 | 0 | 0 |
| - | - | **Total = 21** | **Total = 8** | **Total = 5** |

Our overall findings from this testing of the prototype phase were that:

- For 66 out of 96 citing papers the reference was considered to be highly important by either no assessors or just one and this one assessor was not an expert on any occasion.
- No reference was considered to be highly important to the citing paper by all experts or all reviewers.
- All citing papers where the reference was considered highly important by at least one expert contained three or more citation occasions. See Tables 1&2
- The experts were considerably more conservative than the reviewers in their opinions of the high importance of a reference (mean of papers ticked by experts 6%, mean of papers ticked by reviewers 21%).
- There was substantial variation in the opinions of the assessors for some other parameters, i.e. those included in Section 2 of the Test template. This rendered the collection of these data as unhelpful for our purposes. These categories included whether the citation was noted or reviewed only or included in the text more fully.
